# The Role of Th17 Cells in the Pathogenesis of Behcet’s Disease

**DOI:** 10.3390/jcm6070074

**Published:** 2017-07-21

**Authors:** Yuki Nanke, Toru Yago, Shigeru Kotake

**Affiliations:** Institute of Rheumatology, Tokyo Women’s Medical University, 10-22 Kawada-cho, Shinjuku-ku, Tokyo 162-0054, Japan; toruyago@twmu.ac.jp (T.Y.); skotake@twmu.ac.jp (S.K.)

**Keywords:** Behcet’s disease, IL-17, Th17, regulatory T cells, Th1

## Abstract

Behcet’s disease (BD) is a polysymptomatic and recurrent systemic vasculitis with a chronic course and unknown cause. The pathogenesis of BD has not been fully elucidated; however, BD has been considered to be a typical Th1-mediated inflammatory disease, characterized by elevated levels of Th1 cytokines such as IFN-γ, IL-2, and TNF-α. Recently, some studies reported that Th17-associated cytokines were increased in BD; thus, Th17 cells and the IL17/IL23 pathway may play important roles in the pathogenesis of BD. In this chapter, we focus on the pathogenic role of Th17 cells in BD.

## 1. Introduction

Behcet’s disease (BD) is a polysymptomatic and recurrent systemic vasculitis with a chronic course and unknown cause [1]. BD is characterized by recurrent aphthous stomatitis, uveitis, genital ulcers, and skin lesions. Arthritis is also a common manifestation of BD, and sometimes inflammation is involved in the gastrointestinal tract as well as vascular and central nervous systems. The HLA-B51 gene is closely associated with BD in different ethnic groups. Resent genome-wide studies showed the association of several non-histocompatibility complex (MHC) genes, including IL-10 and IL-23R-IL12 RB 2 genes [2,3]. The pathogenesis of BD is still unclear; in addition to genetic factors, immune dysfunction, and cytokines, viral, and bacterial agents are associated with the exacerbation of the disease. In BD, CD4+ T cells and neutrophils play an essential role in the pathogenesis of the disease. Since IFN-γ and IL-12 from Th1 cells can mediate the inflammatory response between T cells and neutrophils, BD has historically been regarded as a Th1- mediated disease [4,5]. Th17 cells are a novel T cell population that play a major role in autoimmunity. Th17 cell differentiation from naïve CD4+ T cells is facilitated by some cytokines, including IL-1β, IL-6, IL-21, and IL-23. The critical feature of Th17 cells is the expression of Il-17A, IL-17F, IL-6, IL-8, TNF-α, Il-22, IL-26, and the expression of RAR-related orphan receptor (ROR) γ. Recently, the immunopathological effects of Th17 cells in the development of BD were reported. Since IL-17 has been shown to selectively recruit neutrophils to the site of inflammation, abnormalities in the T cell response result in the hyper-reactivity of neutrophils in BD through the production of cytokines such as IL-17 [6]. We review the pathogenic role of Th17 cells in BD in this chapter.

## 2. Th-17 in Mouse Model

In mice, the combination of TGF-β and IL-6 plays an important role in the development of Th17 cells from naïve T cells. Th17 cells and IL-17 play critical roles in the pathogenesis of intraocular inflammation in an animal model of human uveitis [7,8,9]. Anti-mouse IL-17-blocking antibodies suppress intraocular inflammation in experimental uveitis models [10].

The down-regulation of IL-6 [11] and inhibition of the expression of TNF-α [12] improved the inflammatory symptoms in BD mice through the up-regulation of Th17 cells. Foxp3 has been speculated to inhibit Th17 differentiation by antagonizing the function of RORγt, the master transcription factor (mice). Sugita et al. showed that anti-TNF-α blockade may prevent the differentiation of Th17 cells in animal models for BD [13]. γδ Τ cells have also been shown to produce IL-17 and may play a crucial role in experimental uveitis in animal models [9].

## 3. Th17 in Humans

### 3.1. Plasma IL-17 Levels in BD

In humans, IL-1β and IL-23 are required for the development of Th17 cells. Some investigators [14,15,16] reported the ability to produce IL-17A and the percentage of circulating Th17 cells were increased in patients with active BD. Hamzaoui et al. also demonstrated that both the population of Th17 cells and the ability to produce IL-17A were enhanced in active BD, despite the low expression of RORγt mRNA [14].

### 3.2. Increased Circulating Th17 Cell Frequencies are Correlated with Disease Activity

It has been reported that there is a significantly higher frequency of circulating Th17 cells in active BD patients compared with the same patients in remission stages [14]. A positive correlation was noted between C reactive protein (CRP), erythrocyte sedimentation rate (ESR), and the plasma IL-17 level in active BD patients [14]. Some reports showed that the peripheral blood Th17/Th1 ratio was significantly higher in patients with active BD compared with healthy controls [17,18], and that in BD patients with uveitis or folliculitis, the Th17/Th1 ratio was more elevated [15,18]. Thus, they suggested that the balance of Th17 and Th1 cells plays a crucial role in the pathogenesis of BD, especially in the pathogenesis of uveitis and folliculitis. Furthermore, the elevated expression of IL-23p19 mRNA was found in the erythema nodosum (EN)-like lesion of BD [19].

Na et al. [20] reported that IL-17 and IFN-γ expressing CD4+memory T cells were significantly increased in patients with BD compared with healthy controls (HC). In addition, IL-17, IL-23, IL-12/23p40, and IFN-γ in serum and supernatants were significantly elevated in active BD patients compared with HC [20]. IFN-γ-secreting Th17 cells have been found to be elevated in BD patients [20,21]. Thus, BD is associated with a mixture of TH1/Th17 cytokines.

Patients with BD in remission expressed low Th17 levels compared to active BD [14,20,22]. Thus, the population of Th17 cells is correlated with BD activity [16,23].

### 3.3. IL-23–IL-17 Axis

A recent study revealed that IL-23R is essential for the terminal differentiation of IL-17-producing effector T cells in vivo [24]. IL-23 was essential to maintain and to generate Th17 cells, even in the absence of TGF-β [25] The IL-23–IL-17 axis is important for the inflammatory reaction in BD [15]. High levels of IL-17 [14,20] and IL-23 were noted in peripheral blood mononuclear cells (PBMC) from active BD patients [15]. Recombinant IL-23 stimulated the production of IL-17 in CD4+ T cells in BD patients [15]. Recent genetic surveys including GWAS identified IL-23R, IL-12RB2, and IL-10 as BD susceptibility loci [2,3].

### 3.4. The Suppressive Effect of IL-27 on Th17 Cell Differentiation

IL-27 is an essential regulator of the proinflammatory T cell response. In mouse studies, IL-27 plays a negative role in Th17 cell differentiation. Wang C. et al. [26] reported a decreased level of IL-27 in active BD patients. Moreover, decreased IL-27 expression was correlated with uveitis activity in BD patients [26]. The inhibitory effect of recombinant IL-27 (rIL-27) on human Th17 cell differentiation was caused by up-regulation of the expression of interferon regulatory factor (IRF) 8 [26]. Previous studies revealed that the presence of IL-27 may limit Th17-mediated uveitis [27].

It was reported that the expression of IL-21 was elevated in the serum of active BD patients and this promoted Th17 differentiation [23].

## 4. Th17 in Uveitis in BD

It has been reported that IL-23, IFN-γ, and IL-17 both in the sera and aqueous humor showed a significant increase in BD patients with active uveitis compared with BD patients without active uveitis and HC [15]. In addition, increased frequencies of IFN-γ-producing and IL-17-producing T cells in BD patients with active uveitis were reported [15,26]. IL-17 was principally produced by CD45RO+ memory T cells. Thus, the IL23/IL17 pathway is associated with active uveitis in BD patients. It has also been reported that activated CD4+ T cells obtained from BD patients produce TNF-α in vitro. Jiang et al. reported a strong association of rs17375018 in the IL-23R gene with uveitis in BD [28]. Chi et al. showed that IL-12 exerted its inhibitory effect on IL17 through IFN-γ. They also showed that recombinant-IL-23 (rIL23) can promote the production of IL-17 by CD4+ T cells in BD patients [15]. Taken together, the up-regulated IL-17 levels may be associated with the intraocular inflammation of BD patients [15].

### 4.1. Th17 in Skin

A study using antibodies to IL-17A reported that an important population of IL-17+ cells infiltrate the erythema nodosum (EN)-like eruption in BD skin lesions [14]. Simizu et al. also reported that IFN-γ + IL-17-producing cells were dominant and some of them were CD4+ cells in BD-EN [21]. Th17 cells are elevated in circulation and distribution over the skin lesions of BD. Thus, Th17 cells contribute to the pathophysiology of BD.

### 4.2. Th17 in Entero-BD

Gastrointestinal involvement is one of the important complications of BD. Emmi G. et al. [29] reported that T cells at the intestinal mucosal level produce a large concentration of TNF-α, and in the early stage of BD, Th1 and Th17 cells induce inflammation leading to mucosal damage though abnormal and long-lasting cytokine production as well as though both perforin- and Fas-Fas ligand-medicated cytotoxicity. Imamura et al. showed the infiltration of CD4+ and CD8+ T cells in intestinal lesions of BD, along with the expression of mRNAs of proinflammatory and Th1 cytokines/chemokines [28]. In contrast, Ferrante et al. reported that mRNA and the serum level of IL-17 and IL-23 in BD patients with gastrointestinal involvement were not different from those with HC; therefore, a Th1 but not a Th17 response occurs with the gastrointestinal involvement of BD [30]. Thus, more studies are needed to address the pathogenetic role of IL-17 in entero-BD.

### 4.3. Th17 in Neuro-BD

The expression of RORC, which is the master transcription factor of Th17 cells, was reported to be increased in the cerebrospinal fluid (CSF) of patients with neuro-BD [31]. In addition, in the CSF, the Th17/Treg cell ratio was increased [31]. It was reported that there was an elevated level of IL-17 secretion in the sera of BD patients, and the increased expression of transcription factors for Th17 cells was detected in the CSF of BD patients with neurological involvement [31]. Geri et al. detected IL-21- and IL-17A-producing T cells in the CSF, brain parenchyma inflammatory infiltrates, and intracerebral blood vessels from patients with active BD and CNS involvement [23]. The stimulation of CD4+ T cells with IL-21 increased Th17 and Th1 differentiation and decreased Treg cells [23]. On the other hand, Diresskeneli et al. [32] reported that IL-17 was not detectable in either the serum or CSF of neuro-BD patients. Thus, the pathogenetic role of IL-17 in neuro-BD remains controversial.

## 5. Polymorphisms

It was reported that Th1- and Th17-related cytokines and signaling molecules participated in BD pathogenesis [33,34,35]. Some studies demonstrated that polymorphisms of Th17-related cytokines and receptors such as IL-17F, IL-23R, and IL-23 A were associated with BD susceptibility in Chinese and Korean populations [36,37,38]. STAT4 is essential for the expansion of Th17 cells activated by IL-23. Functional studies indicated that the risk of single nucleotide polymorphisms (SNPs) in the STAT4 gene involved in the pathogenesis of BD may affect the expression of STAT4 and the production of IL-17 [39]. The haplotype of IL17 A showed a positive association with the intestinal BD risk, where those of IL23R protected against disease development. The interactions of specific IL17A, IL23 Rs, and STAT4 SNPs modulate susceptibility to intestinal BD in the Korean population, suggesting the potential significance of the IL-17/23 axis in the pathogenesis of intestinal BD [40].

## 6. Plasticity

Recent studies showed that the plasticity of Th17 and Th17 cells means that they have the ability to produce Th1 (IFN-γ) or Th2 (IL-4)-type cytokines under inflammation [41,42]. Th17 cells can turn into IFN-γ-expressing T cells in mouse Th1 disease models, which are named Th1-like cells, IFN-γ-expressing Th17 cells, or Th17/Th1 cells. The expression of RORC—the master transcription factor of Th17—is not fixed in T cells, and the plasticity of Th17 cells was noted in murine models in vivo [28]; some reports have applied this conception to human diseases [43,44]. Geri et al. reported that the frequencies of both IL-17+CD4+ T cells and IFN-γ+CD4+ T cells were elevated in the CSF compared to the PBMC in BD patients [23]. Th17 and Th1 cells may be involved at different stages in inflammation, and Th17 cells were generated more than Th1 cells during inflammation. The increase in Th17/Th1 and Th17/Treg ratios is correlated with the extent of inflammation. As in many other inflammatory diseases, in BD, plasticity exists between Th1, Th17, and Treg cells during inflammation in the peripheral circulation and at inflammatory sites [45]. The decreased levels of Th17 in remission BD compared with active BD could be explained by a conversion of Th17 cells into Treg cells. The differentiation of Tregs into Th17 cells was involved in the down-regulation of FoxP3 expression and the suppressor function. Foxp3 has been suggested to inhibit Th17 differentiation by antagonizing the RORγt function [46].

## 7. Treatment with Cyclosporin A

Cyclosporin A (CsA) is effective for reducing the frequency and severity of intraocular inflammation in BD. Chi et al. demonstrated that CsA has an effect on both IL-17 and IFN-γ production in vitro and in vivo. In vitro, it was shown that CsA inhibited IL-17 production from peripheral blood mononuclear cells (PBMC) of BD patients. In vivo, the amelioration of intraocular inflammation in BD was accompanied by the suppression of both IL-17 and IFN-γ production after CsA administration [22]. Taken together, it is suggested that the efficacy of CsA on uveitis in BD is through inhibiting of IL-17 and IFN-γ production.

### 7.1. Treatment with Antibodies to IFN-α

Type I IFNs including IFN-α could inhibit IL-17 production by PBMC. Recombinant IFN-α has been used to treat BD [41]. In vitro experiments showed that IFN-α does not directly modulate the Th1/Th17 balance in BD, but rather promotes a regulatory Th1 response through IL-10 secretion [47]. IFN-α activity was mediated via STAT2 phosphorylation [48]. IFN-α was also able to up-regulate the gene expression of IL-27—a negative regulator of Th17 cells [49].

### 7.2. Treatment with Anti-TNF-α Therapy

TNF-α has been found in BD patients [4]. Anti-TNF-α therapy suppresses effector T cell differentiation in BD patients with uveitis [13,50]. It was reported that the production of IL-17 by polarized Th17 cell lines exposed to infliximab in vitro or fresh CD4+ T cells from BD patients being treated with infliximab was reduced, and the RORγt in T cells was also reduced. Thus, TNF-α is required for TH17 differentiation in BD. CD4+ T cells exposed to anti-TNF-α therapy may convert into Treg cells.

Anti-TNF-α therapy-induced Treg cells from BD patients suppressed the activation of target T cells [13]. Thus, the Th17/Treg balance may be important for the pathogenesis of inflammation in BD [33,48].

### 7.3. Treatment with Antibodies to IL-17 A

IL-17A has an important role in acute attacks of not only eye disease but also oral ulcers, genital ulcers, and articular symptoms [14,15,16]. IL-17A from patients with active BD can elevate the expression of adhesion molecule mRNA. Treatment with antibodies to IL-17A suppressed the production of adhesion molecules [14,51]. Thus, therapeutic modalities attempting to evaluate new approaches to eliminate the overactivities of IL-17A and/or the IL-23/IL-17 pathway may clarify the pathological importance of Il-17A and Th17 cells in BD patients.

## 8. Conclusions

BD is predominated by Th1 and Th17 immune responses. Th17 cells are associated with the active inflammation of BD. Thus, Th1/Th17-type immune responses and the IL-23-IL-17 axis are important for the inflammatory reaction and have a pathologic role in BD.

## Abbreviation

BD: Behcet’s disease
